# Supplementary material for: Rice β-Glucosidase 4 (Os1βGlu4) Regulates the Hull Pigmentation via Accumulation of Salicylic Acid
Source: Int J Mol Sci. 2022 Sep 13;23(18):10646. doi: 10.3390/ijms231810646 (PMC9504040; doi:10.3390/ijms231810646)
Supplement: Supplementary file 1 [file ijms-23-10646-s001.zip › Table S1.pdf]

Table S1. The primer used in this study.

Primer name	Primer name Primer sequence (5′-3′)
**Map-based cloning**	
RM6581-F	GCTCGAGAACCATGTGGGATAGC
RM6581-R	CCCACCTCGCTTCTCCTTCTCC
RM7650-F	TTACAGTCAAGGTCTCGCTCTCG
RM7650-R	TCGGAAGACCACTCACTCTCTCC
RM3632-F	GGTGAAATACACGGTGACTTGAGG
RM3632-R	TGGGTTCTTCTTCAGCAGTGTCC
RM1261-F	ATGGTAGAGACACAAGTCCATGC
RM1261-R	GACAAATTGGTGTAGGTGAAGG
1-20-F	AAGGAGAAGTGGAGGTTAAGATAGGG
1-20-R	GTTAATTTACTGACCGGCCATGC
1-21-F	GTGATGCTTTCGTCGTCGTTGG
1-21-R	GGAGGACAAGAACAAGAACAAGAACG
**CRISPR/Cas9 vector**	
U6AL-*Osbg1*-F	AAACTGCTAGATGCTACTgttttagagctagaaat
U6AL-Os-*bg1*-R	AGTAGCATCTAGCAGTTTCCggcagccaagccagca
U-F	CTCCGTTTTACCTGTGGAATCG
gR-R	CGGAGGAAAATTCCATCCAC
Pps-F	TTCAGAggtctcTctcgACTAGTATGGAATCGGCAGCAAAG
Pgs-R	AGCGTGggtctcGaccgACGCGTATCCATCCACTCCAAGCTC
**Complementation and GFP vector**	
F	ATGGGGAGCACGGGGCGC
R	GCCTTCGTTCTGCAGCAACC
L-F	ctgtacaaggagctcggatccATGGGGAGCACGGGGCGC
L-R	caggtcgactctagaggatccGCCTTCGTTCTGCAGCAACC
Promotor-F	TGAGTGCACCACCATTCAGATAA
Promotor-R	GAGGCGTGAAGAAGACTTGTGC
Com/pro-F	gagctcggtacccggggatccTGAGTGCACCACCATTCAGATAA
Com/pro-R	catGAGGCGTGAAGAAGACTTGTGC
Com/CDS-F	agtcttcttcacgcctcATGGGGAGCACGGGGCGC
Com/CDS-R	cttgcatgcctgcaggtcgacCTAGTTCATGTCAGCTTTGTTCTCAGC
**GUS vector**	
Pro-F	tggctgcaggtcgacggatccTGAGTGCACCACCATTCAGATAA
Pro-R	tcttagaattcccggggatccGAGGCGTGAAGAAGACTTGTGC
