# Supplementary figures and images for: Rice β-Glucosidase 4 (Os1βGlu4) Regulates the Hull Pigmentation via Accumulation of Salicylic Acid
Source: Int J Mol Sci. 2022 Sep 13;23(18):10646. doi: 10.3390/ijms231810646 (PMC9504040; doi:10.3390/ijms231810646)

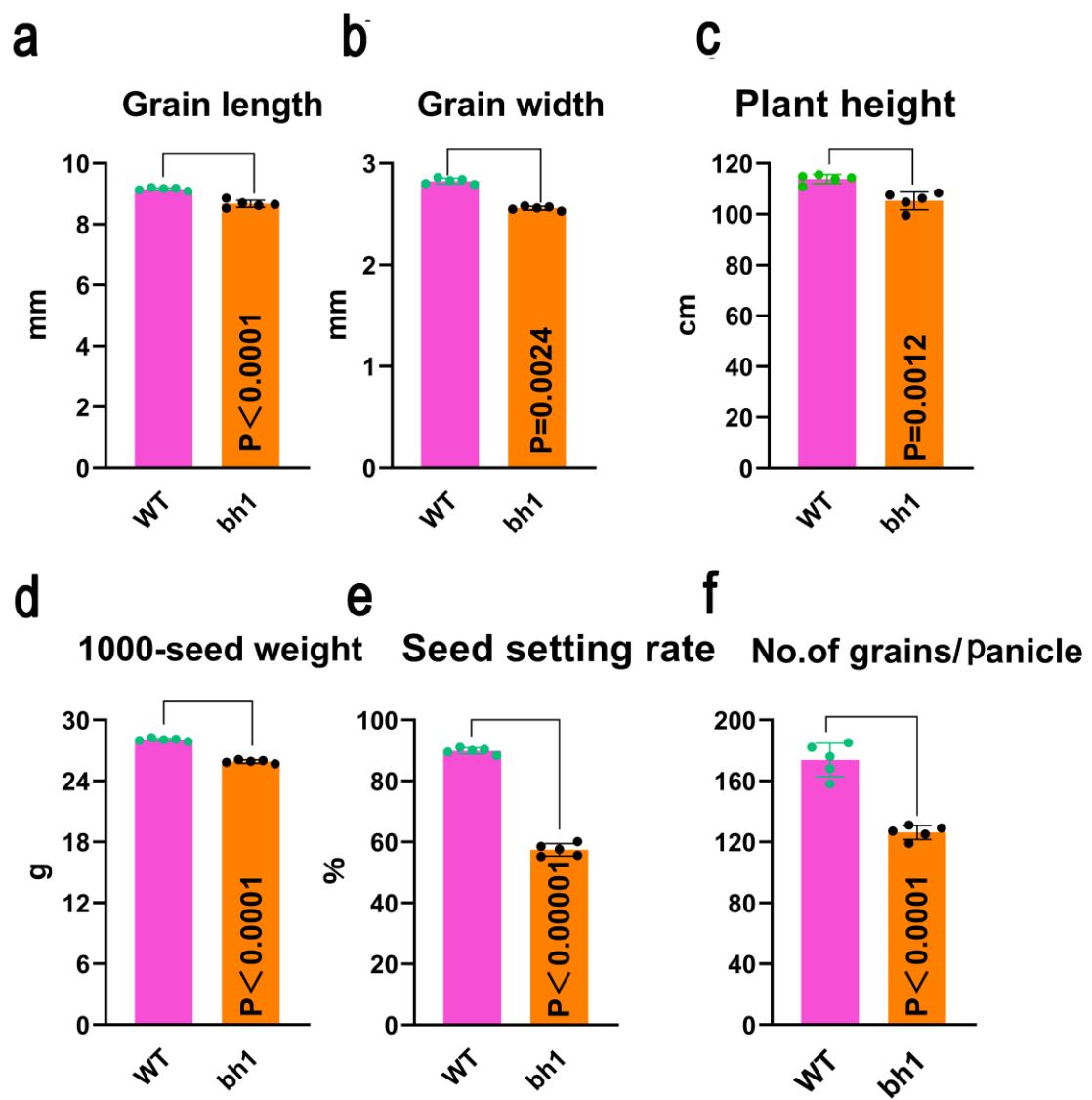

Figure S1.

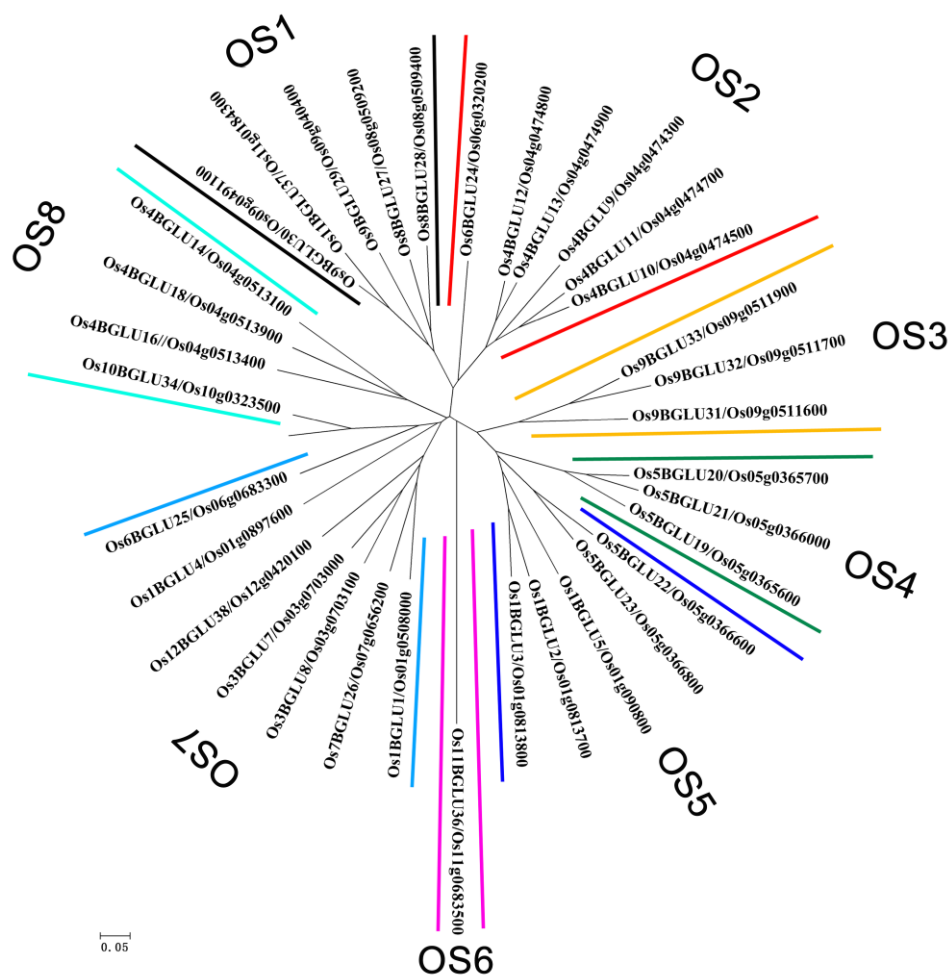

Figure S2.

Supplement: Supplementary file 1 [file ijms-23-10646-s001.zip › Figures S1 and S2.pdf]
